# The Survey of Personal and National Identity on Cell Phone Addicts and Non-Addicts

**Published:** 2018-01

**Authors:** Seyyed Salman Alavi, Maryam Ghanizadeh, Mohammad Reza Mohammadi, Soroush Mohammadi Kalhory, Fereshteh Jannatifard, Ghazal Sepahbodi

**Affiliations:** 1 Psychiatry and Psychology Research Center, Tehran University of Medical Sciences, Tehran, Iran.; 2 Master of Psychology, Kerman University of Medical Sciences, Kerman, Iran.; 3 Young Researchers and Elite Club, Roudehen Branch, Islamic Azad University, Roudehen, Iran.; 4 Ministry of education, Tehran, Iran.

**Keywords:** *Addict*, *Cell Phone*, *Identity*

## Abstract

**Objective:** Smart phones have rapidly become an integral, and for some, an essential communication device worldwide. The issue of identity has always been a subject of interest among psychologists. The present study was conducted to compare personal and national identity and their subscales between cell phone addicts and non-addicts.

**Method** **:** In this cross-sectional study, 500 student cell phone users from various universities in Tehran were recruited using stratified sampling. Participants completed cell phone addiction questionnaires including Mobile Phone Problematic Use Scale (MPPUS), Cell Phone Dependency Questionnaire (CPDQ), Personal Identity Development Questionnaire, Extended Objective Measure of Ego Identity Status (EOMEIS) and National Identity Questionnaire. Then, the subscales of these instruments were analyzed using SPSS Version 20.

**Results:** Results of this study revealed significant differences between cell phone addicts and non-addicts in the scores of national identity, personal identity, and most subscales, except for some subscales (P<0.05). In addition, a negative and significant relationship was found between personal and national identity and cell phone addiction (r=-0.35, -0.33, respectively).On the other hand, after controlling for the confounder variables, we found that national identity had an effect on cell phone addiction(OR=0.05, CI=0.92-0.98).

**Conclusion: **The results of this study indicated that cell phone overuse may be correlated with defects in some aspects of national and personal identity.

The subject of identity has been an issue among psychologists. Dependency to cyberspace among adolescents, especially in recent decades, has led to an increase in anxiety and depression, as well as identity crises rooted in modern global developments(1). Because the majority of virtual space users are adolescents, a group dealing with rapid physiological, bodily and psychological changes, it should come as no surprise that this group are more vulnerable to the consequences of cell phone misuse, as they are passing the stages of identity formation and entering adulthood.

The relationship of cell phone possession and cell phone use with psychosocial and identity development was investigated using Erikson’s Psychosocial Theory and Marcia’s Adolescent Identity Paradigm(2).Marcia (1991) considers puberty to be a stage of destruction in the course of identity formation. Therefore, new devices including electronic media, computers, the internet, cell phones, and their misuse can influence identity construction(3).

Previous research has found identity status to be related to internet use, music, and television viewing preferences (4-7).

Based on previous research, it appears that adolescents distinctively use technology to satisfy their social, emotional, and developmental needs, which are in turn associated with their current identity formation. Thus, if identity status is related to internet use, music, and television, it would seem logical that these relations extend to cell phone use as well (2).

Since self-identity or the perceived value of cell phone use might be a predictor of frequency of cell phone use, it would determine or predict dependence or implication of cell phone usage(8).Therefore, it is of paramount importance to determine the pattern of cell phone use and assess the correlation of cell phone addiction with national and personal identity to define the psychological aspects of pathological cell phone use. In the future, we will be faced with a dilemma arising from cell phone addiction and overuse in developing countries including Iran, which can affect our adolescents and their mental health. The present study aimed at examining personal and national identity between cell phone addict and non-addict university students in Tehran by controlling for the impacts of demographic variables such as gender, age, amount of cell phone use, and marital status. It is hypothesized that national and personal identity has an impact on cell phone addiction.

## Materials and Methods

The research method of the present study, which was conducted in 2017, was cross-sectional to determine the association of national and personal identity and the related subscales with cell phone addiction and assess the best predictors based on demographic variables. In social science, a cross-sectional study is a type of study that analyzes data collected from a population, or a representative subset at a special point in time, which is called cross-sectional data(9). 

The statistical population of this study was recruited duringfall2016 and summer 2017. The students who took part in this study were from different universities in Tehran.

Using quota sampling, participants were selected from 4universities in Tehran (University of Tehran, Tehran University of Medical Sciences, Kharazmi University, and Islamic Azad University). First, the total number of students in each university was calculated separately. Then, based on the calculated sample size (500 students) and in proportion to each university, samples were selected using quota sampling and according to the calculated proportion. To calculate sample size, confidence interval (CI) and study power were assessed (95% and 80%, respectively).

Inclusion criteria were as follow: Being a student (freshman, sophomore, etc.), using a smart phone at least once a day in the past year, and using it at least an hour per day. The participants with psychological difficulties, severe physical problems, and those who were under drug therapy or psychotherapy during the past year were excluded.

*Instruments*

The following tools were used in the present research:

1)Demographic Questionnaire, This questionnaire surveys such data as age, marital status, gender, education level, hours of mobile phone use per day, time of use, and the reason for use in each day(eg, calling, texting, social networking).

2) Cell Phone Dependency Questionnaire (CPDQ): This 20- item self-report questionnaire was designed and developed by Toda et al. (2004). The items reflect typical behaviors of behavioral addiction, especially cell phone addiction. Participants responded to each item on a 5-point Likert scale, with a higher score indicating stronger tendency towards cellular phone dependency. This instrument provided different aspects of cell phone dependency phenomenon. Toda et al. demonstrated that reliability (Cronbach’s alpha) of CPDQ was .86 and external reliability(test–retest) was also reported to be satisfactory(10).

Alavi et al. (2014) noted that the Persian version of CPDQ has acceptable validity and reliability. They extracted 3factors including salience, overuse of cell phones, and compulsive use of SMS via factor analysis method. Internal consistency (Cronbach’s alpha) of the CPDQ was 0.88(11).

3)Cell Phone Pathological Use Scale(MPPUS):This 27- item questionnaire was designed by Bianchi and Phillips (2005) based on a 5-point Liker scale; it evaluates many symptoms, such as cell phone addiction, withdrawal symptoms, destructive effect on health, and social, economic, and vocational status. Cronbach’s alpha was reported to be α =0.91for this scale (12).

Alavi et al. reported that MPPUS has acceptable reliability (Cronbach’s alpha=0.91) with adequate factor models to assess the extent of problems caused by the misuse of cell phones in Iranian populations(13). 

4)Diagnostic Interview: Clinical interview, based on DSM-IV-TR criteria for an impulse control disorder (ICD) not otherwise specified (NOS), was performed for all the samples by an expert psychologist, who was educated in the diagnosis of ICD, especially in behavioral addiction. Alavi et al. (2016) reported that internal consistency and test-retest reliability of this interview were 0.55 and r = 0.4 (p<0. 01), respectively(14).

5) National Identity Questionnaire (Iranian): This questionnaire, which was developed by Sirous Ahmadi in 2007, includes 27 questions and 5 subscales, which are as follow: (1) membership in a national group, (2) homeland defense, (3) national heritage, (4) general features of national group, and (5) others’ viewpoints on the national group. All items of this questionnaire have been developed based on a 5-point Likert scale. The validity and reliability of National Identity Questionnaire has been reported to be adequate, and its reliability, according to Cronbach’s alpha coefficient, has been reported to be0.92(15, 16)

6).Extended Objective Measure of Ego Identity Status (EOM-EIS): This scale was developed by Bennion and Adams in 1986 and assesses4 identity aspects including diffusion, foreclosure (premature), moratorium (delayed), and achievement, based on Marcia’s theory. This questionnaire includes 64 questions based on a Likert scale. Aghasoltani et al. reported that Cronbach’s alpha coefficients of the questionnaire were0.72, 0.86, 0.67, and 0.76 for 4domains of diffusion, foreclosure, moratorium, and achievement identities, respectively (1).

Rahiminezhad (2015) found that both ideological and interpersonal identity of EOM-EIS questionnaire had a 4-factor structure as follows: (1) foreclosure, (2) identity achieved, (3) moratorium, and (4) diffused identity. The convergent-divergent validity of the 4-identity statuses yielded significant correlations between the same identity statuses of ideological and interpersonal identity, ranging from 0.38 to 0.65. The internal reliability of the 4 scales of the 2ego identities was0.46 for identity moratorium and diffusion in ideological identity, and correlations ranged from 0.53 to 0.76for rest of them(17).

MPPUS, CPDQ, National and Personal Identity Questionnaires, as well as Demographic Questionnaire were filled out by all participants. Then, a clinical interview based on behavioral addiction was performed for each participant to diagnose cell phone addiction. Finally, after collecting and scoring the questionnaires, the subscales of both National and Personal Identity Questionnaires were analyzed in both groups, based on clinical interview and cut- off point of CPDQ and MPPUS. We used Pearson’s correlation coefficient and multiple logistic regressions in SPSS Version 20.

## Results

A total of 500 students participated in this study and were divided into 2groups of cell phone addicts and non-addicts subsequent to the execution and analysis of CPDQ, MMPUS, according to the participants’ sex.

According to the results, the mean and standard deviation of the age of the sample were 27.9 and 6.9, respectively. Also, the mean and standard deviation of amount of smart phone use in each work shift were 2.18 and 1.4, respectively. In addition, according to cut- off point of clinical interview, CPDQ, and MMPUS, 75% of the participants were non-addicts and 25% were diagnosed as cell phone addicts. (Table 1)

Pearson’s correlation coefficient was calculated to assess the association between personal and national identity and the subscales with cell phone addiction. The findings revealed a significant and inverse relationship of personal identity, national identity, and some subscales (such as general characteristics of national group and national heritage) with cell phone addiction. However, this significance was not present in other factors of national identity (membership in national group, other’s point of view regarding national group, and homeland defense). (Table 2)

Table 3 demonstrates the results of the multiple logistic regression for cell phone addiction. The main variables that influenced smart phone addiction were age, sex, duration of cell phone usage (in hours), and national identity.

Age significantly decreased the chance of cell phone addiction (OR = 0.87, 95% CI, 0.8-0.95).Moreover, based on the results of the logistic regression, sex decreased the odds of smart phone addiction (OR = 0.28, 95% CI, 0.11-0.72).In addition, duration of cell phone use increased the chance of cell phone addiction (OR = 2.1, 95% CI, 1.2-15.9), and national identity decreased the odds of cell phone addiction (OR=0.95, CI=0.92-0.98).

Personal identity and education were not significant in the equation, and other national identity factors did not seem to affect the odds of cell phone addiction.

## Discussion

The present study was conducted to compare personal and national identity in cell phone addict and non-addict students and find the association of cell phone addiction with personal and national identity. Based on the findings of this study, there was a significant difference (P < 0.05) between the 2 groups in the average scores of national identity, personal identity, and some subscales of national identity, such as membership in national group and homeland defense. By comparing the 2groups, it was found that as addiction to cell phone grows to more severe degrees; the denial of personal and national identity is more frequently observed. Based on Funk and Buchman theory, exposure to media and cyberspace might influence the behavior and opinions of individuals, and thus their overuse could have long term negative impacts, leading to a decrease in empathy, religious beliefs, and national values(18).

Furthermore, according to Gruenhage, Vingatesh, and Vitalarie, an misusing or overusing cyberspace may cause extreme loneliness and seclusion, leading to a decrease in social interaction and disordered identity, and ultimately, a reduction in social skills(19). In another research, the findings showed the impacts of internet, computer games, and cyberspace on a number of users who had been continuously using the cyberspace. Their results showed a deterioration of visual problems, neck ache, back ache, development of introversion and isolation, development of aggressive attitudes and social aversion, feelings of religious and national alienation, and harming family economy(1). According to another study by Maleki and Abbaspour, the scores of the national identity of persons who do not use media (internet, mobile phones, and satellite television channels) are significantly higher than those of persons who use these communication devices(20).

**Table 1:** Summary of the Demographic Information of Participants According to Features of the Demographic Questionnaire and Diagnostic Criteria for Cell Phone Addiction

**Variable**		**Diagnosis of cell phone addiction**		**Percentage**
Sex	Yes		Male	13.6
			Female	84.4
	No		Male	23.2
			Female	76.8
Age	Yes		18-25 years	73.2
			26-30 years	21.4
			31 years and more	5.4
	No		18-25 years	31.8
			26-30 years	27.3
			31 years and more	40.9
Marital Status	Yes		Married	21.4
			Single	78.6
	No		Married	54.5
			Single	45.5
Education	Yes		Freshman	3.6
			Bachelor’s degree	33.9
			Master’s degree	41.1
			PhD and higher	21.4
	No		Freshman	1.1
			Bachelor’s degree	25.6
			Master’s degree	54
			PhD and higher	19.3
Duration of cell phone usage	Yes		Less than 1 year	1.1
			1 to 2 years	4.3
			More than 2 years	94.6
	No		Less than 1 year	2.8
			1 to 2 years	5.7
			More than 2 years	91.5
Frequency of cell phone use	Yes		Every day	66.1
			Every other day	23.2
			2 days per week and more	10.7
	No		Every day	31.8
			Every other day	35.8
			2 days per week and more	32.4

**Table 2:** Pearson’s Correlation Coefficient of Personal and National Identity and the Subscales and Addiction to Cell Phones

**Variables**	**Mobile addiction**	**P- value**
National identity	-0.33^*^	P<0.001
Membership in the national group	0.08	P>0.05
General characteristics of the national group	-0.24^*^	P<0.01
National heritage	-0.29^*^	P<0.01
Other’s point of view regarding the national group	0.09	P>0.05
Homeland defense	0.10	P>0.05
Personal identity	-0.35^*^	P<0.01

**Table 3:** Odds Ratios (95% CI) for Cell phone Addiction in Participants (n = 500)

**Variables**	**Univariate analysis**			**Multivariate analysis**		
	**OR(crude)**	**CI (95%)**	**P-value**	**OR(adjusted)**	**CI (95%)**	**P-value**
Age	0.82	0.77-0.89	0.00	0.87^*^	0.8-0.95	0.002
sex	0.52	0.24-1.2	0.09	0.28*	0.11-0.72	0.009
Duration of cell phone usage(in hours)	2.6	1.7-4.1	0.00	2.1*	1.2-3.4	0.004
Marital status	0.22	0.11-0.45	0.00	0.51	0.21-1.2	0.14
National identity	0.95	0.98-1.1	0.08	0.95*	0.92-0.98	0.05
Personal identity	1.1	0.98-1.2	0.09	0.62	0.7-2.1	0.06
Education	0.8	0.55-1.1	0.26	0.88	0.98-1.6	0.09

OR adjusted: Odds Ratio

CI: Confidence Interval

In another study, findings revealed a significant difference between the scores of national and religious identity and mental health among 3 groups of cyberspace users; they found that religious, national identity, and mental health were significantly different between computer users and other groups, meaning that overuse of the cyberspace negatively affects mental health. However, the mentioned results have not been reported to be significant in comparison of personal identity(21).Huang has noted that cell phone addicts and non-addicts are significantly different in their identity status(22). Nevertheless, the results of the present study revealed no significant differences in the national heritage in the national identity scores between the two groups. This might be due to the influence of factors other than cyberspace on national heritage among Iranians. All individuals in a society will be mobilized to protect their country in case of serious threats; similarly, many Iranians admire their national heritage including poets, kings, and Nowrouz celebrations; however, it seems that such factors as cyberspace may have negative effects on these national and cultural values.

Moreover, the findings indicated a significant and reverse association between national and personal identity scores and cell phone addiction (P < 0.05), except for the factor of general characteristic of national group and homeland defense. The relationship between cell phone addiction and such factors as social seclusion, education failure, lack of efficacy, social support, identity disorder, anxiety, and self-reliance was reported to be significant in a study(11),which means that with the increase in the severity of smart phone addiction causes social isolation, occupational and educational failure, identity disorders, and lack of self-esteem, and self-reliance(22).

Furthermore, in their study, Hafeznia, Kaviani- Rad Karimipour, and Taherkhani reported that development of globalization processes in the form of communication technology, such as the internet or other communication devices, is related to national identity; this means that with the increase in the use of technology, there is a decrease in national values (23). In addition, in another study, the association between technology and the acquisition of personal identity, especially in the youth, has been considered to be of major importance (22). A lack of relationship between national heritage and homeland defense comes from the interference of number of mediating and moderating factors, such as cultural conditions or economic status, which have not yet been determined.

The obtained results indicated that age significantly decreased the occurrence of cell phone addiction (OR = 0.87) (positive effect), signifying that by per year increase in age, the possibility of occurrence of cell phone addiction reduced by 0.13. Some other studies found sociological commonalities. In a study, young adolescents had a higher tendency towards cell phone use and were at more risk of smart phone addiction(24).Walsh et al. reported that self-identity at an early age is the predictor of cell phone use and that there is robust correlation among cell phone dependency, self-identity, and being female (8). Furthermore, De-Sola Gutierrez et al. (2016) found that an excessive use of cell phones has been correlated with personality traits including neuroticism, self-esteem, self-identity, and self-image (25). However, the exact causes of the association of personal and national identity with cell phone addiction have not yet been discovered. Perhaps, the lack of national belonging per se makes a person susceptible to overuse his/her cell phone and become a cell phone addict. Alternatively, the lack of belonging to national identity could be the effect of cyberspace or cell phone addiction, which means that those who become addicted to their cell phones may experience negative changes in their personal and national identity. Due to many complications, this issue needs further investigation. However, identity (such as personal, national, religious etc.) of cell phone addicts in general is more susceptible than that of non-addicts. As stated in the previous research, cell phone, its brand, and facilities play crucial roles in developing the self-identity of young adults(26).Our findings revealed that duration of cell phone use (in hours) increased the chance of cell phone addiction (OR = 2.1). Similarly, Zurkefly, Baharudin (2009), and Choliz (2012) reported that students who used cell phone for 2 or 5 hours in a day were diagnosed as cell phone addicts (14, 27, 28). Alavi et al. (2015) stated that the proposed diagnostic criterion for cell phone addiction is its long-time use (29). Furthermore, a study found a positive correlation between time per day spent on cell phones and cell phone addiction(11), which means that students who overuse cell phones are more at risk of cell phone addiction.

Cell phones have become important communication devices, can be used for academic purposes, are entertaining, and can be used to exchange information. Thus, further studies should be conducted to survey the direct association of identity and cell phone dependence and to precisely determine whether cell phone addiction leads to a lack of desire for personal or national identity. To sum up, a greater trend towards the cyberspace is correlated with higher transformation in the dimensions of identity among adolescents. Also, as adolescence is considered a period of identity formation, parents need to discuss the possible disadvantages of the cyberspace and cell phone use with their children (30).

## Limitation

Despite the benefits of this study, some limitations in the research evidence have been identified. First, our findings did not apparently indicate whether identity characteristics in this study preceded the development of cell phone addiction behavior or were they the outcome of smart phone use. Second, the process of sample collection did not allow generalization of the findings to the non-college population. 

Another possible limitation of the present study was its data, which were collected over a very short time. Moreover, using numerous scales, such as CPDQ, MPPUS, and National or Personal Identity Questionnaire, had their restrictions.

## Conclusion

Considering that the national or personal identity was associated with problematic cell phone use, there was an inverse relationship between national or personal identity with cell phone addiction. 

Nevertheless, complexity of cell phone addiction stems from multiple factors, such as educational, cultural, economic, mental health, and social factors, which could impact cell phone addiction.

Eventually, in addition to the mentioned arguments, cell phone addiction has been recently considered as a social dilemma, and the number of clients who present this problem to psychological clinics is gradually increasing. Therefore, all perspectives of this issue and its negative and positive outcomes should be thoroughly determined. The results of this study should be presented to Iran’s cultural authorities to provide them with real insight regarding the issue and to generate the ability to develop proper strategies based on scientific premises.
